# 

**Published:** 1878-02

**Authors:** 


### BOOKS AND PAMPHLETS RECEIVED.

The Science and Art of Surgery; being a Treatise on Surgical Injuries, Diseases and Operations. By John Eric Erichsen, F. R. S., F. R. C. S., etc., etc. Revised by the author from the seventh and enlarged English edition. Illustrated with eight hundred and sixty-two engravings on wood. 2 vols. Philadelphia: Henry C. Lea. 1878. 8 vo.; leather, pp. 946 each vol. Price, $10.50. For sale by Jansen, McClurg & Co.

Pneumono-Dynamics. By G. M. Garland, M. D., etc. New York: Hurd & Houghton. Boston: H. O. Houghton & Co. Cambridge: The Riverside Press. 1878. 8vo.; paper, pp. 155.

A Guide to Therapeutics and Materia Medica. By Robert Farquharson, M. D., etc. Enlarged and adapted to the U. S. Pharmacopœia. By Frank Woodbury, M. D., etc. Philadelphia: Henry C. L. Lea. 1877. 8vo.; cloth, pp. 410. Price, $2.00. For sale by Jansen, McClurg & Co.

The Elements of Therapeutics; a Clinical Guide to the Action of Medicines. By Dr. C. Binz. Translated from the Fifth German Edition, and edited with additions in conformity with the British and American Pharmacopœias, by Edward I. Sparks, M. B. Oxon., etc., etc. New York: Wm. Wood & Co. 1878. 8vo.; cloth, pp. 347. Price, $2.00. For sale by Jansen, McClurg & Co.

Public Hygiene in America; being the Centennial Discourse delivered before the International Medical Congress, Philadelphia, September, 1876. By Henry I. Bowditch, M. D., with extracts from correspondence from the various States, together with a Digest of American Sanitary Law. By Henry G. Pickering, Esq. Boston: Little, Brown & Co. London: Trubner & Co. 1877. 8vo.; cloth, pp. 498. Price, $2.50. For sale by Jansen, McClurg & Co.

Cyclopædia of the Practice of Medicine. Edited by Dr. H. Von Ziemssen, Professor of Clinical Medicine, Munich, Bavaria. Vol. XIV. Diseases of the Nervous System and Disturbances of speech, by Professors A. Eulenburg, H. Nothnagel, H. Von Ziemssen, F. Jolly, A. Kussman and Dr. J. Bauer. Translated by Drs. E. Buchanan Baxter, Alexander Morrison, David Lincoln, Geo. B. Shattuck, Samuel Webber, J. Haven Emerson, John A. McCreery. Albert H. Buck, Editor American Edition. New York: Wm. Wood & Co. 1877. 8vo.; cloth, pp. 893.

Transactions of the Twenty-Second Annual Session of the Michigan Dental Association; held at the Dental Department of the University, Ann Arbor, Oct. 9th, 10th and 11th, 1877. Published by order of the Association. Ransom & Randolph, Toledo, Ohio. 8vo.; paper, pp. 88.

Transactions of the Canada Medical Association. Tenth Annual Meeting, Montreal, Sept. 12th and 13th, 1877. Vol. I. Montreal: Printed by Lovell Printing and Publishing Company. 1877. 8vo.; paper, pp. 244.

A Case of Syphilitic Aphasia. By L. P. Yandell, Jr., M. D. Printed from the *Medical News* of Dec. 8, 1877.

What is Modern Homeopathy? By S. W. Wetmore, M. D., etc., etc. Reprint from *American Observer.*

Exposition of Facts. By A. T. Garnet, M. D.

An Address Read at the First Meeting of the American Academy of Medicine. By the Secretary, R. Lowry Sibbart, A. B., M. D., in Philadelphia, Sept, 6, 1876. On the Necessity of an Organization which shall Encourage a Higher Standard of Qualifications in the Medical Profession in the United States. Carlisle, Pa., 1877.

Do Valor Therapeutico das Injeçcoes Hydricas Subcutaneas. Pelo Dr. Moncorvo, Membro de Acad. de Med. do Rio de Janeiro, etc. etc. Extrahido do *Progresso Medico.*

Do Emprego do Chlorate de Potassa ha Diarrhéa das Criançcas. Pelo Dr. Moncorvo, Membro Correspondente de Sociedad de Medicina de Pariz.

Westerman & Co. Medical Catalogue.

Bulletin Mensuel des Nouvelles Publications. Christern. Libraire à New York, Etats Unis d'Aémrique.

Bulletin des Publications Nouvelles. De G. Masson, Editeur Librairie de L'Académie de Médecine. No. 21, December, 1877.

1878. Mansill's Almanac of Planetary Meteorology and New System of Science. By Richard Mansill, Author of "Cohesive Attraction and Formation of Worlds," "Earthquakes and Volcanic Eruptions," "Universal Changes in Natural Elements," "The New Law of Gravitation," "Six Titles in Natural Law," etc., etc. Price, 50 cents. R. Crampton, Publisher, Rock Island, Ill.

Annual Address. Reprint from Vol. 2 Gynecological Transactions, 1877. By the President, Fordyce Barker, M. D., of New York.

Ninety-fifth Annual Catalogue of the Medical School of Harvard Universtiy (Boston). 1877–'78. (Reprint from the Catalogue of the University).

The Narcotic Effect of Morphia on the New-born Child when Administered to the Mother in Labor. By Walter R. Gillette, M. D. Reprint from *The American Journal of Obstetrics and Diseases of Women and Children.* Vol. X, No. IV; October, 1877.

Higher Medical Education, the True Interest of the Public and of the Profession. An Address Introductory to the 112th Course of Lectures in the Medical Department of the University of Pennsylvania. Delivered Oct. 1, 1877. By William Pepper, A. M., M. D., etc. Published by order of the Board of Trustees, and at the request of the Medical Class. Philadelphia: Collins, Printer. 1877.

Scarlatina in Chicago, Particularly the Epidemic of 1876-7. By Chas. W. Earle, M. D. Read before the Illinois State Medical Society.

On the Nature, Origin and Treatment of Puerperal Fever. By W. T. Lusk, M. D. Extract from the transactions of the International Medical Congress. Philadelphia: September, 1876.

Ovariotomy by Enucleation. By Julius F. Miner, M. D. Extract from the transactions of the International Medical Congress. Philadelphia: September, 1876.

Contributions to the History of Medical Education and Medical Institutions in the United States of America. 1776—1876. Special Report prepared for the United States Bureau of Education by N. S. Davis, A. M., M. D. Washington: Government Printing Office. 1877.

Spinal Irritation in Children as Related to True and False Arthropathies. By V. P. Gibney, M. D., etc. Reprint from the transactions of the American Neurological Association for 1877. New York: 1877.

Obituary of Joseph Warren Freer, M. D., President and Professor of Physiology and Microscopic Anatomy in Rush Medical College, Chicago.

A study of Nine Hundred and Sixty-five Cases of Chronic Pulmonary Diseases. By F. H. Davis, of Chicago, Ill. Extracted *Trans. Amer. Med. Ass'n.* Philadelphia: 1877.

An Inquiry into the General Pathology of Scurvy. By Charles Henry Rolfe, M. A., M. D., Cantab. Reprint from the *Lancet.* London: H. K. Loomis, 136 Gower street, W. C. 1877.

The Influence of High Altitudes on the Progress of Phthisis. By Chas. Denison, A. M., M. D. Ext. Trans. International Medical Congress. Philadelphia: September, 1876.

Character of Edward Hammond Clarke; the Man and the Physician. A Sermon preached in the West Church, Boston, Sunday, Dec. 9, 1877. By C. A. Bartol. Boston: 1878.

Chronic Inversion of the Uterus. Address by Prof. James P. White, M. D., Vice President of the International Medical Congress. Philadelphia: 1876.

---

List of Practitioners who Passed the Examination of the Illinois State Board of Health, held at Springfield, Jan. 10th, 1878: G. V. Black, Jacksonville, Morgan; Ezra Brown, Sciota, McDonough; M. A. Bently, Plattville, Kendall; R. U. Chapman, Kappa, Woodford; J. B. Clark, Seymore, Champaign; C. A. Feltman, Salem, Madison; F. A. Hall, Cordova, Rock Island; C. R. House, La Crosse, Hancock; E. B. Hughes, Smithfield, Fulton; T. M. Johns, Taylorville, Christian; W. H. Lanoix, Quincy, Adams; John McGhee, Dillon, Tazewell; T. B. Norwell, Harkins Corners, Peoria; A. J. Overholt, Loami, Sangamon; J. M. Roy, Burnt Prairie, White; G. H. Rue, Ivesdale, Champaign; A. R. Spriggs, Rinard, Wayne.

List of Practitioners who Passed the Examination of the Illinois State Board of Health, held at Champaign, Dec. 20, 1877: Jerome Arnold, Wilmington Brese P. O., Greene; A. M. Bird, Mason City, Mason; John Beasley, Mount Pleasant, Union; W. H. Burtnett, Camargo, Douglas; S. L. Chapin, Downs, McLean; C. A. Dean, Salem, Marion; Louis Loda, Hartsburg, Logan; E. E. McKain, New Hebron, Crawford; W. D. Matney, Harvel, Montgomery; Duncan Miller, Peoria, Peoria; A. P. Rockey, Prairie Bird, Shelby; Y. D. Scales, Manchester, Scott; S. S. Spees, Tuscola, Douglas; J. J. Starkey, Waynesville, De Witt; T. R. Scott, Attila, Williamson; T. B. Straus, Gibson, Ford.

### ANNOUNCEMENTS FOR THE MONTH.

### SOCIETY MEETINGS.

Chicago Medical Society—Mondays, Fub. 4 and 17.

Chicago Society of Physicians and Surgeons—Mondays, Feb. 11 and 24.

### CLINICS.

MONDAY.

Eye and Ear Infirmary—2 to 4 p. m., by Prof. Holmes and Dr. Hotz—2 p. m., Prof. Jones.

Mercy Hospital—2 to 3 p. m. Surgical, by Prof. Andrews.

Rush Medical College—1:30 p. m. Medical, by Dr. Bridge.

County Hospital—8 p. m. Necropsy, by Dr. Danforth.

Woman's Medical College—3 p. m. Surgical, by Prof. Owens.

TUESDAY.

County Hospital—1:30 p. m. Medical, by Prof. Bevan; 2:30 p. m. Surgical, by Dr. Bogue.

Mercy Hospital—2 p. m. Medical, by Prof. Hollister.

Eye and Ear Infirmary—2 p. m. Prof. Jones.

WEDNESDAY.

County Hospital—1:30 p. m. Gynecological, by Prof. Fitch; 2:30 p. m. Ophthalmological, by Dr. Montgomery.

Mercy Hospital—2 p. m. Eye and Ear, by Prof. Jones.

Rush Medical College—4 p. m. Diseases of the Chest, by Prof. Ross.

THURSDAY.

Mercy Hospital—2 p. m. Medical, by Prof. Davis.

Rush Medical College—1:30 p. m. Neurological, by Prof. Lyman.

Eye and Ear Infirmary—2 to 4 p. m. Operations by Prof. Holmes and Dr. Hotz.

FRIDAY.

Mercy Hospital—2 p. m. Medical, by Prof. Davis.

County Hospital—1:30 p. m. Medical, by Prof. Ross; 2:30 p. m., Surgical, by Prof. Gunn.

Woman's Medical College—10 p. m. Ophthalmological, by Dr. Montgomery.

SATURDAY.

Rush Medical College—2 p. m. Surgical, Prof. Gunn.

Chicago Medical College—2 p. m. Surgical, by Prof. Andrews and Isham; 3 p. m., Diseases of the Chest, by Prof. Johnson.

Woman's Medical College—12 m. Gynecological, by Prof. Fitch; 3 p. m. Dermatological, Dr. Maynard.

Special Clinics daily, from 2 to 4 p. m., at the South Side Dispensary, and at the Central Free Dispensary.

For schedule of lectures at the colleges, apply to the college janitors.

